# Assessment of eating disorder symptoms, compulsive exercise, body dissatisfaction and depression in Swedish national team gymnasts, with a one-year follow-up

**DOI:** 10.1007/s40519-024-01667-3

**Published:** 2024-07-12

**Authors:** Klara Edlund, Niklas Forsberg, Henrik Källberg, Anna Melin

**Affiliations:** 1 Division of Psychology, Department of Clinical Neuroscience, Karolinska Institutet, 17177 Stockholm, Sweden; 2 Musculoskeletal and Sports Injury Epidemiology Center, Department of Health Promotion Sciences, Sophiahemmet University, Stockholm, Sweden; 3 Stockholm, Sweden; 4 Department of Sports Science, Linnaeus University, Växjö/Kalmar, Sweden

**Keywords:** Eating disorder symptoms, Body dissatisfaction, Drive for thinness, Compulsive training, Depression

## Abstract

**Purpose:**

The purpose of this study was to explore changes in symptoms of eating disorders, compulsive exercise, and depression, between two assessments 12 months apart, among elite gymnasts.

**Method:**

Factors related to the development of mental health symptoms in male and female Swedish national team gymnasts were investigated using baseline and 1-year follow-up scores in two subscales of the Eating Disorders Inventory 3; drive for thinness and body dissatisfaction, two subscales of the Compulsive Exercise Test; avoidance and rule-driven behavior and exercise for weight control, and the Montgomery-Åsberg Depression Rating Scale-Self report (MADRS-S). Linear mixed models were used to investigate the influence of drive for thinness, exercise for weight control, avoidance and rule-driven behavior, and MADRS-S on body dissatisfaction.

**Results:**

Body dissatisfaction increased from baseline to the follow-up assessment, while drive for thinness and depression remained stable. Symptoms of eating disorders and depression were generally low in this group of elite gymnasts at both assessments. Drive for thinness, exercise for weight control, and symptoms of depression were associated with body dissatisfaction.

**Discussion:**

Our findings indicate that there were no significant changes over time in eating disorders and depression symptoms but significant associations with body dissatisfaction. Furthermore, we found independent effects of drive for thinness, exercise for weight control and symptoms of depression for body dissatisfaction.

**Supplementary Information:**

The online version contains supplementary material available at 10.1007/s40519-024-01667-3.

## Introduction

Eating disorders are conditions that profoundly impact health and performance in athletes [1], and elite-level sport participation has been suggested to increase the risk of developing eating disorders [2]. Some studies have reported higher prevalence of eating disorder symptoms among athletes compared to non-athletes [e.g., [3]]. Eating disorders have been suggested to be under-recognized, under-reported, and under-diagnosed among male athletes [4], due to a variety of reasons related to differential presentation of symptoms, secretiveness or shame around behaviors, and sex-related stigma [4, 5]. Marí-Sanches and co-workers [1] advocate for more research about eating disorders to make epidemiological and etiological advances for effective improvement of prevention, and to establish treatment strategies tailored for athletes [1].

Athletes follow intensive training schedules, and many perceive pressure to maintain a low body weight and leanness for the purpose of maximizing performance [6]. Competing in esthetic sports may increase the susceptibility to eating disorder psychopathology, compared to non-esthetic sports, due to the perception that body weight and shape significantly alters performance in sport [7]. Sport-related pressures to maintain a certain physique can result in excessive training (e.g., training for too long, too often, or too intensively), as well as pathological eating behaviors (e.g., dietary restriction, self-induced vomiting) to alter body shape and weight [6]. Gymnastics is an esthetically judged sports with a common belief that judges are influenced by the gymnasts’ body composition, and an increased risk of eating disorders in esthetical sports has been reported [2]. There are four different disciplines in gymnastics (Supplement A), where three are Olympic disciplines (artistic and rhythmic gymnastics and trampoline gymnastics) and the fourth discipline is rapidly growing as a competitive elite-level sport genre in Europe (team gymnastics).

There are a handful publications since 1996 on eating disorders in artistic and rhythmic gymnastics, respectively, and, to our knowledge, no publications for trampoline gymnastics or Team gym. Although there are differences between the four gymnastics disciplines of how the sports are executed, the similarities regarding sport-specific body ideals, early specialization, tight and revealing leotards for training and competing as well as rigorous and intense training routines are similar. The Swedish Gymnastics Federation is the fourth largest sport federation in Sweden in terms of the number of members. Team gymnastics is the largest discipline and one of the overall fastest growing sports in Sweden and in Europe where team gymnastics is now represented in the official International Federation of Gymnastics (FIG; https://www.gymnastics.sport/site/) European championships. Although team gymnastics also is aesthetically judged the body ideal seam to vary between the gymnastic disciplines with regard to low weight with a lean body composition vs. a more muscular body composition. These differences have, to our knowledge not been studied, but are relevant to differentiate between risk behaviors and symptom presentation to identify gymnasts in need of assessment and discipline-specific interventions. As pointed out by Sundgot-Borgen, almost 30 years ago [7], there is a need to learn more about how risk factors and the etiology of eating disorders differ between and within sports. Further, there is a need to integrate factors such as training environment and sport climate into the athlete health perspective. Recent media coverage indicates that an unrelenting need to succeed within elite sport can create detrimental and harmful environments where performance and results are given priority at the expense of athletic welfare [8]. Further, the understanding of normalization of eating disordered behaviors and maltreatment of gymnasts in the training environment needs more attention. This to fully understand gymnasts’ apprehension of ill-health and unhealthy training environments where disordered eating may be encouraged [9].

The recent meta-analysis by Chapa and co-workers [2] indicate that female athletes report lower levels of body dissatisfaction compared to non-athletes, although both groups report similar levels of eating disorder symptoms. Indications of higher levels of eating disorder symptoms such as drive for thinness, restrictive eating, and loss of control of eating is reported among athletes in esthetic and leanness demanding sports compared to non-esthetic/leanness sports.

In a study, Villa and co-workers [10] analyzed dietary intake and body composition in a group of elite-level competitive rhythmic gymnasts (age 9–18) from Spain. Their main conclusion was that teenage gymnasts do not have a sufficient daily energy intake, explaining the gymnasts’ low body weight and putting them at a higher risk of developing low energy availability (LEA). Many athletes are driven by strong pressure to achieve optimal performance which may contribute to altered energy intake and exercise energy, resulting in problematic LEA [11–13]. The 2023 consensus statement by the International Olympic Committee (IOC) [13] refines the understanding of the outcomes of problematic LEA exposure causing Relative Energy Deficiency in Sports (REDs), where also the athletes mental health is taken into greater account. Psychological factors contributing to LEA and mental health consequences have previously been highlighted [11]. Depressive symptoms and affective disorders [14–16], exercise dependence [15, 17], anxiety related to injury and recovery, sport-specific issues such as difficulty coping with weight requirements [18, 19], and the development of eating disorders [20] are all aspects of mental health outcomes associated with problematic LEA and REDs, beside health decrements such as reproductive dysfunction, poor bone health and sport injuries [13].

Compulsive exercise consists of maladaptive compensatory behaviors and serves as a strategy for emotion regulation—often closely linked to eating disturbances, especially in females [21]. Compulsive exercise is characterized by an inability or unwillingness to cut down or stop the behavior despite adverse health consequences [22]. In this pursuit, athletes may become malnourished and enter a state of LEA and REDs [13]. Compulsive exercise is linked with poorer treatment outcomes for eating disorders and should therefore be assessed to ensure that training is not a compensatory behavior for dietary intake [19]. Research suggest that high-performance athletes represent a complex sub-group of the eating disorders population, and there are indications that a different conceptualization of compulsive exercise is needed in this population [23].

There are ongoing discussions about whether sports participation is a risk- or protective factor for eating disorders [2]. Research is mixed, with some studies suggesting that athletes have higher mean levels of eating disorder psychopathology compared to non-athletes, while other studies suggest the opposite effect or no differences [2]. In a study of Icelandic elite athletes, at the very national top level (*n* = 755, representing 20 different sports), Kristjánsdóttir and co-workers [24] reported that 17.3% of the study sample analyzed reported severe or moderate body image concern, reaching 39.4% in esthetic sports. Although it is unclear if athletes have higher or lower mean levels of eating disorder psychopathology compared to non-athletes, previous studies have provided valuable insights into the prevalence of eating disorders among elite athletes and have highlighted the need for early detection, prevention, and intervention strategies [12]. Further, there is a lack of knowledge as far as long-term understanding of gymnasts’ health.

There is one longitudinal study assessing eating disorders in female college athletes (*n* = 193), from their competitive years to retirement from their sport (6 years later; [25]). The results indicated that retirement does not result in immediate remittance of eating concerns among female athletes and many of the athletes continued or develop eating disorder symptoms. The authors suggest addressing healthy body image and nutrition when athletes are competing as imperative to assist prevention and intervention efforts that may alleviate eating disorder symptoms [25]. Thus, longitudinal studies are needed to understand the aetiology of health problems related to eating disorders in gymnasts. Studies that identify risk and prognostic factors are required to develop health promotion, prevention strategies, which ultimately can reduce the burden of eating disorders in gymnasts.

The aim of this study was to explore changes in symptoms of eating disorders, compulsive exercise, and depression over 12 months among Swedish national team gymnasts. Further, to analyze associations between drive for thinness, compulsive exercise, and depression with body dissatisfaction in this group of elite gymnasts.

## Methods

### Study design

The present study was a longitudinal observational study of factors related to the development of eating disorder symptoms in elite gymnasts. This design provided the opportunity to study longitudinal changes over a 12-month period, and account for intra-individual variability in our linear mixed models.

### Participants and procedures

The Swedish national team of gymnastics and gymnasts invited to train at the national team’s annual winter camp (*n* = 193), were invited to participate in the study, all competing on an international junior or senior level representing the four international competitive gymnastic disciplines. Five gymnasts declined participation, 11 did not attend camp, hence 177 (88%) participated in this study endorsed by the Swedish Gymnastics Federation. Only gymnasts that were present and filled out the questionnaires at both camps were included in the present study (*n* = 94). Follow-up assessment took place 12 months after baseline assessment, again at the annual winter camp. The measure was completed in a lecture hall at the sport facility. There was a traditional classroom test setting where the gymnasts were seated with space between their seats and were asked not to talk to one another. Only the research staff was present when the measures were filled out, both to assist with the questionnaires but also to ensure privacy while filling out the survey.

Written informed consent was required by all participating gymnasts—and for gymnasts younger than 18 years of age parental consent was required. After retrieving informed consent all gymnasts attending the national team’s winter camp filled out a self-report questionnaire (pen & paper) onsite at camp. The study was approved by the Swedish Ethical Review Authority (dnr: 2016/084) and performed in accordance with the Declaration of Helsinki. There was no compensation for the gymnasts for participating in the study.

### Measures

#### Background and demographic questions

A short form assessing demographics such as age, gender, height, weight was developed for this study. Further, training load and meal pattern was assessed.

#### Eating Disorders Inventory 3 (EDI 3)

The Eating Disorders Inventory 3 (EDI 3) is a self-report questionnaire intended to assess attitudes, emotions and behaviors typically associated with eating disorder symptomatology and has been validated to be an appropriate screening instrument for disturbed eating behavior in non-clinical settings [23]. Two subscales, drive for thinness (7 items) and body dissatisfaction (10 items), of the Swedish version of the EDI 3 were included in the present study [26]. The drive for thinness and body dissatisfaction subscales were used to identify gymnasts with eating disorder symptoms. The subscales consisted of a 6-point Likert scale with responses ranging from “always” to “never”. Items were scored as following: 0 for the two responses indicating no eating disorder symptoms, and 1, 2, 3 or 4 points accordingly for scores indicative of more severe eating disorder symptomatology.

Based on a study by Nyman-Carlsson and co-workers [27] where Swedish norm data were presented, a score of drive for thinness ≥ 12 or body dissatisfaction ≥ 19 was used to categorize gymnasts being at risk of eating disorders. Mean scores presented in the study for normal controls (*n* = 648) was 6.2 for drive for thinness and 15.9 for body dissatisfaction. These scores are referred to as norm data from the general population. Earlier studies have reported an adequate sensitivity (69–80%), specificity (70–79%; 24), and internal consistency for the two subscales: drive for thinness (*α* = 0.85), and body dissatisfaction (*α* = 0.87; 25). Our calculation regarding internal consistency for drive for thinness resulted in a Cronbach’s alpha equal to 0.63 which is associated with questionable consistency and for body dissatisfaction Cronbach’s alpha was 0.78 which is considered acceptable consistency.

### Compulsive Exercise Test

The Compulsive Exercise Test (CET; [28]) comprised 24 self-report items that are designed to assess the core cognitive, behavioral, and emotional features of compulsive exercise. Items are rated on a 6-point Likert type scale from 0 (never true) to 5 (always true) and two subscales were used: avoidance and rule-driven behavior and exercise for weight control. Subscale scores are reflecting the mean of score per relevant item within the subscale. The CET has previously demonstrated good psychometric properties in non-clinical samples with excellent concurrent and convergent validity, and Cronbach’s alpha coefficients ranging from 0.72–0.88 [26]. The subscales exercise for weight control and avoidance and rule-driven behavior were included in this study since they have been reported to be most suitable for an athletic population [28]. Higher scores on the Compulsive Exercise Test are indicative of greater pathology and have been reported to have adequate validity (factor analysis) and internal consistency (exercise for weight control: *α* = 0.82, avoidance and rule-driven behavior: *α* = 0.87; 27). Cronbach’s alpha among a sample of more than 4000 Swedish university students has confirmed excellent internal consistency (exercise for weight control: *α* = 0.83, and avoidance and rule-driven behavior: *α* = 0.90) [28]. Our calculation regarding internal consistency for CET resulted in a Cronbach’s alpha equal to 0.70 which is on the limit for acceptable.

#### Montgomery-Åsberg Depression Rating Scale-Self report (MADRS-S)

The Montgomery-Asberg Depression Rating Scale (MADRS-S; [29]) is one of the most widely used rating instruments for screening, diagnosis and measuring the severity of depression. MADRS-S focuses on the psychological symptoms of depression during the past three days (e.g., sadness, tension, and pessimistic thoughts). This scale consists of 9 items; each item is rated on a 0–6 scale, resulting in a maximum total score of 54 points, with higher scores indicative of greater depressive symptomology. The MADRS-S scoring instructions indicate that a total score, ranging from 20–34 indicates “moderate depression”, and a score of > 35 indicates “severe depression”. An improvement of two points or more on the MADRS-S is considered clinically relevant. Previous psychometric study [30] reported the intraclass correlation coefficient of the MADRS-S as acceptable for unipolar depression, ranging between 0.83 and 0.86. Overall, the MADRS-S has been found to have sound psychometric properties [31].

### Statistical analysis

All data were prior to analysis checked for normality. Age, BMI and exercise for weight control was normally distributed, all other variables were violating normal distribution. Comparisons between years were analyzed using paired t-test for normally distributed variables and Wilcoxon signed rank test with continuity correction for the remaining variables. Results are either reported as mean ± standard deviation, or median (25th percentile – 75th percentile).

Linear mixed models were used to investigate the influence of drive for thinness, exercise for weight control, avoidance and rule-driven behavior, and MADRS-S on body dissatisfaction scores, using the subject ID as the random effect to account for variability within subjects that is not explained by the fixed effects, using the lme4 package [32]. In the first modeling step, drive for thinness, exercise for weight control, avoidance and rule-driven behavior, and depression were regressed exclusively on the outcome body dissatisfaction in three steps. First without adjusting for any control variables, second by adjusting for sex and age, and in the final step adjusting for sex, age, and discipline (categorized as team or individual). Finally, drive for thinness, exercise for weight control, avoidance and rule-driven behavior and depression was regressed simultaneously on body dissatisfaction in the same three steps as in the isolated variable analyses. Please find the equation for the model in Supplement B.

Results from the linear mixed models are presented as the ß-coefficient (95% CI). Analysis was carried out using R version 4.2.1 [33].

## Results

### Descriptive statistics

In total, 94 gymnasts completed the questionnaire both at baseline and at the 1-year follow-up of which 41 (44%) were males and 53 (56%) were females, of which 53 (56%) were team gymnasts and 41 (44%) individual gymnasts.

Gymnasts participating in team gymnastics were older than the individual gymnasts [17.0 (16.0–20.0) years vs. 15.0 (14.0–19.5) years, *p* = 0.006], while there was no difference in age between females and males [16.0 (15.0–21.5) vs. 16.0 (14.0–18.5) years, *p* = 0.276]. Based on age-adjusted BMI, one gymnast (1%) reported in the BMI-range that is considered “thinness” (-2 SD) at baseline and reported a change to the range of “underweight” (- 1 SD) at follow-up. At baseline, 9% (*n* = 8) were overweight (+ 2 SD for gymnasts < 18 years, or BMI ≥ 25 for gymnasts > 18 years), and 12% (*n* = 11) at follow-up. The reason for using − 1 SD and − 2 SD is that − 1 SD is considered as underweight, and − 2 SD is considered thinness according to the World Health Organization.

### Changes in exercise for weight control, body dissatisfaction, avoidance and rule-driven behavior and depression

Table 1 presents differences between baseline and the follow-up assessment after 12 months, for all participants. 4 gymnasts did not report height and/or weight at both assessments. For the remaining 90 gymnasts, BMI increased from baseline to the follow-up (21.38 ± 2.33 vs. 22.06 ± 2.13, *p* < 0.001); see Table 1. Exercise for weight control and body dissatisfaction increased from baseline to follow-up, while the scores for drive for thinness, avoidance and rule-driven behavior and depression remained stable (Table 1).

**Table 1:** Longitudinal changes for total sample (*n* = 94). Data are presented as either mean ± SD or median (25th–75th percentile)

Variable	Baseline	Follow-up	*p*-value
BMI	21.38 ± 2.33^(*n*=90)^	22.06 ± 2.13^(*n*=90)^	< 0.001
EWC	1.82 ± 0.88	2.12 ± 0.79	0.002
ARDB	3.00 (2.04–3.55)	2.86 (2.29–3.86)	0.178
DT	2.50 (0.25–5.00)	3.00 (1.00–6.00)	0.094
MADRS-S	7.00 (4.00–12.00)^(*n*=90)^	8.00 (4.00–12.00)^(*n*=90)^	0.248
BD	5.00 (1.00–9.00)	7.00 (3.00–13.00)	< 0.001

*ARDB*  Avoidance and Rule-Driven Behaviors from the Compulsive Exercise Test, *BD* body dissatisfaction from the Eating Disorders Inventory, *BMI*  body mass index, *DT*  Drive for Thinness, *EWC*  Exercise for Weight Control variable from the Compulsive Exercise Test, *MADRS-S*  The Montgomery-Asberg Depression Rating Scale

At baseline and follow-up, 4 (4%) and 11 (12%) of gymnasts, respectively, scored high on the drive for thinness scale; see Table 2. The corresponding number of gymnasts scoring high on body dissatisfaction was 3 (3%) and 8 (9%) at baseline and follow-up, respective. In total, 5 (5%) gymnasts reported elevated drive for thinness and/or body dissatisfaction scores at baseline and 15 (16%) at follow-up. At baseline, 4 gymnasts did not fill out the MADRS-S satisfactory. At baseline, 2 gymnasts (2%) reported moderate depression and at follow-up 6 (6%) reported moderate or severe depression.

**Table 2:** Number of individuals scoring high on respective scale at baseline, follow-up, and at both data collections, thereby classified as experiencing ED symptoms and depression

	Baseline	Follow-up	Both years
DT	4 (4%)	11 (12%)	2 (2%)
BD	3 (3%)	8 (9%)	2 (2%)
MADRS-S	2 (2%)^(*n*=90)^	6 (6%)	2 (2%)

*BD*  body dissatisfaction, *DT*  drive for thinness, *MADRS-S*  The Montgomery-Asberg Depression Rating Scale

### Mixed model

In Table 3, using a mixed model, exercise for weight control, avoidance and rule-driven behavior, drive for thinness and depression were associated with body dissatisfaction when considered exclusively. All variables remained significant when adjusting for age and sex; and for age, sex, and discipline (team or individual), meaning that all independent variables were associated with body dissatisfaction, when analyzed exclusively.

**Table 3:** Estimates from separate models

Variable	Crude (*β* (95% CI))	Adjusted(1) (*β* (95% CI))	Adjusted(2) (*β* (95% CI))
DT	0.72 (0.54–0.86)***	0.65 (0.52–0.79)***	0.67 (0.54–0.81)***
EWC	2.69 (1.79–3.60)***	3.10 (2.18–4.06)***	3.06 (2.16–4.02)***
ARDB	2.16 (1.27–3.06)***	1.74 (0.87–2.60)***	1.67 (0.79–2.54)***
MADRS-S	0.42 (0.27–0.56)***	0.34 (0.20–0.48)***	0.34 (0.21–0.48)***

Outcome variable: BD This table shows *ß*-values and 95% CI for each of the variables DFT, EWC, ARDB and MADRS-S included exclusively in the model with each individual as the random effect. Crude involves only the variable of interest, Adjusted(1) is adjusted for sex and age, Adjusted(2) is adjusted for sex, age and discipline

*ARDB*  Avoidance and Rule-Driven Behaviors from the Compulsive Exercise Test, *DT*  Drive for Thinness, *EWC*  Exercise for Weight Control variable from the Compulsive Exercise Test, *MADRS-S*  The Montgomery-Asberg Depression Rating Scale

^***^p < 0.001

When drive for thinness, exercise for weight control, avoidance and rule-driven behaviors and depression were regressed simultaneously on the outcome variable body dissatisfaction, drive for thinness and depression explained part of the variance of body dissatisfaction (*p* < 0.05), where exercise for weight control and avoidance and rule-driven behavior did not in the crude model (*p* ≥ 0.05); see Table 4. However, drive for thinness, depression and exercise for weight control could explain part of the variance in body dissatisfaction when adjusting for age and sex; and age, sex and discipline (team gymnastics or individual disciplines), indicating that there were independent effects of drive for thinness, exercise for weight control and depression on body dissatisfaction.

**Table 4:** All estimates from the same model

Variable	Crude (*β* (95% CI))	Adjusted(1) (*β* (95% CI))	Adjusted(2) (*β* (95% CI))
DT	0.58 (0.43–0.72)***	0.51 (0.37–0.65)***	0.54 (0.40–0.68)***
EWC	0.82 (− 0.02–1.65)	1.32 (0.56–2.31)**	1.22 (0.37–2.11)**
ARDB	0.66 (− 0.08–1.40)	0.43 (− 0.28–1.13)	0.27 (− 0.43–0.95)
MADRS-S	0.31 (0.20–0.42)***	0.26 (0.16–0.37)***	0.27 (0.16–0.37)***

Outcome variable: BD This table shows *ß*-values and 95% CI for DFT, EWC, ARDB and MADRS-S included simultaneously in the model with each individual as the random effect. Crude involves only the variables of interest. Adjusted(1) adjusted for sex and age. Adjusted(2) adjusted for sex, age and discipline

*ARDB*  Avoidance and Rule-Driven Behavior from the Compulsive Exercise Test, *BD* body dissatisfaction, *DT*  Drive for Thinness, *EWC*  Exercise for Weight Control variable from the Compulsive Exercise Test, *MADRS-S*  The Montgomery-Asberg Depression Rating Scale

*p* < 0.05*, *p* < 0.005**, *p* < 0.001***

## Discussion

To our knowledge, this is the first study of athletes in elite-level national team gymnasts with regard to eating disorder symptomatology and mental health. The current study including gymnasts from all Swedish national teams in gymnastics (artistic gymnastics, rhythmic gymnastics, team gymnastics, and trampoline gymnastics) were included and the study was conducted using longitudinal design over a 12-month time-period. The main findings was that there was little change over time in eating disordered behaviors and depression. As far as eating disorder symptoms, this is in line with the longitudinal study by Thompson and co-workers, [25] indicating that spontaneous remission of eating disorder symptoms are scares. Based on previous studies, we can speculate that the results may reflect athletes under-reporting eating disorders and depression due to factors such as stigma and concerns about whether symptoms of eating disorders and depression could have a negative impact on their career, such as affected chances of making the team for future competitions [3, 5, 34]. Worth noticing is that the prevalence of mental health problems (e.g., depression) in the general population of, e.g., Swedish university students has been reported to be high [35, 36] and that positive effects of training and exercise for mental health problems may also come into play in the gymnast sample explaining the low prevalence of eating disorder symptoms and depression [37].

### Eating disorder symptoms, body dissatisfaction, compulsive exercise, BMI, and changes over time

In the present study, 16 gymnasts (17%) reported eating disorder symptoms, such as drive for thinness (14%) and body dissatisfaction (10%), at one or both assessment time points. Like previous findings, the majority of athletes reporting eating disorder symptoms were females (94%) [21]. However, it might be under-recognized among males when only using the body dissatisfaction and drive for thinness subscales as the preferred body image among male gymnasts might to a greater extent not be thinness but rather leanness and muscularity [21]. Thus, the used measure “drive for thinness” in the EDI 3 addresses restrictive eating, fear of weight change and preoccupation with food and weigh—and not just, as the name suggests, drive for thinness with low body weight and a lean body composition. Furthermore, the gymnasts in the present study reported low levels of moderate or severe depression (6%), and the majority reported a body mass within the normal range. Symptoms of depression commonly co-occur with eating disorder symptoms which may in part explain that levels of depression did not change over time in our sample. As far as body dissatisfaction, a possible explanation that gymnasts report lower levels of body dissatisfaction than controls from the general population could be that their training routines prevent them from engaging in compensatory exercise behaviors to control their weight. The link between body dissatisfaction, compulsive exercise, and depression in the general population, among university students, revealed that 38% of participants (*n* = 4263) reported at least mild concern with their shape, which is higher than in the present study of gymnasts [38] and can serve as an example of differences between athletes and the general population.

### Prediction of body dissatisfaction

Body dissatisfaction have in numerous studies, been reported to be a central factor for both the development and maintenance of eating disorders. Body dissatisfaction is differentiated from eating disorders and compulsive/excessive exercise behaviors and therefore it is important to highlight the need to consider them as separate constructs in research and clinical practice [39–41]. Further, body dissatisfaction is prevalent across various populations, including individuals with and without diagnosed eating disorders or excessive exercise behaviors [39]. Yet, body dissatisfaction has not, to our knowledge, been addressed as an outcome variable with the notion that other factors such as compulsive exercise and eating disordered behaviors could drive increased levels of body dissatisfaction which in turn could further manifest- or increase the risk of an eating disorder. This may be of importance in a clinical perspective when working with gymnasts since interventions for body dissatisfaction may focus on improving body image through cognitive-behavioral techniques, body acceptance practices, and addressing underlying self-esteem issues [41]. Treatment for eating disorders and excessive exercise often involves a broader range of approaches, including nutritional counseling, psychotherapy, medication, and addressing comorbid conditions [41].

Although the scores in the present study were low, the body dissatisfaction score increased among all gymnasts from baseline to follow-up, as did exercise for weight control, supporting previous reports of co-occurrence of body dissatisfaction and behaviors for weight control. We identified that drive for thinness, both constructs of compulsive exercise (exercise for weight control and avoidance and rule-driven behaviors) and symptoms of depression were associated with body dissatisfaction. Our results indicate that there are independent effects of drive for thinness, exercise for weight control and symptoms of depression for body dissatisfaction, with the results adjusted for sex, age, and discipline. Although our sample did not report high prevalence of eating disorder symptoms, the longitudinal design of our study suggests a potential increase of symptoms over time through the increase in body dissatisfaction and exercise for weight control scores. The implications as far as clinical work with gymnasts, our results suggest close monitoring of key behaviors that may increase the risk of developing more pathological behaviors such as comments on weight and shape, dietary restriction and close awareness of symptoms of mental health problems. There is a need for awareness of differences as far as symptoms in the sport-specific environment compared to the general population where athletes seem to have a higher barrier seek help due to stigma and sport-specific barriers [42]. We suggest that close attention is paid to sensitive developmental periods in young gymnasts such as puberty and weight change. We see the need for longitudinal assessments over time with regard to risk factors and behaviors for the development of eating disorders. This is essential to identify athletes at risk of developing an eating disorder and to establish sound interventions for treatment and prevention.

### Future directions

Like most studies assessing risk and eating disorder symptoms we have used validated questionnaires. There are indications that athletes tend to under-report symptoms and behaviors when using self-report measures when compared to clinical interviews. Future studies are therefore suggested to not only study development over time using longitudinal designs with an age and gender matched control group, but also to include clinical interviews conducted by licensed personnel. Further, the assessment of leanness and muscularity is also suggested in upcoming studies along with analyses of differences regarding these constructs between the different disciplines. Although these suggested procedures are thorough and time consuming, prospective studies integrating clinically valid diagnostic methods could provide important data on changes in risk behaviors for eating disorders, from adolescence to adulthood. Further, focus on early identification of risk behaviors among athletes are warranted along with the development of sound strategies for early identification of eating disorder symptoms along with effective interventions for treatment and prevention.

### Strengths and limitations

Strengths of the current study included all Swedish gymnasts of all four disciplines on the national teams were invited to participate with the study, the longitudinal design, and the equal number of female and male participants. The response rate was high, both at inclusion and follow-up one year later. Limitations include self-report and possible risk of under-reporting of symptoms due to data being collected at camp. Another limitation was the lack of power analyses which could have affected the results due to a risk of the sample not being powered for the executed analyses. The reason for the lack of power analysis prior to the data collection was that we could not affect which gymnasts were invited, and thus not the sample size. Due to the unequal sample sizes for the different disciplines between-group analyses could not be conducted which could be considered a limitation of the study.

Further, the suboptimal of internal consistency observed for drive for thinness (Cronbach’s alpha = 0.63) might indicate that the scale might not be a good measurement for the intended purpose of this study, and thus being a limitation. Despite the questionable measure, we did not see changes in the variable from baseline to follow-up, and it could explain part of the variance in body dissatisfaction with and without adjusting for covariates. It is however still possible that some of the 7 items in the scale were driving the association that we observed.

When evaluating the ß-coefficients from the linear mixed models, caution is warranted as the explanatory variables are based on different scales. Drive for thinness score and depression are measured as the total score of the scales, while the constructs of compulsive exercise represent the mean score of each item in the scales. Hence, the coefficients represent what a 1 unit increase for drive for thinness or depression is expected to have on body dissatisfaction, while a 1 unit increase in exercise for weight control score represents an increase of 5 points for the total score (prior to division of 5) and 7 points, respectively, for the total avoidance and rule-driven behavior score (prior to division of 7).

## Conclusions

The main findings were that there were no significant changes over a 12-month period of time in reported symptoms of eating disorders and depression among Swedish national team gymnasts. Further, drive for thinness, exercise for weight control, and symptoms of depression were associated with body dissatisfaction indicating that indirect measures of eating disorder characteristics could be useful to target increased risk of ill-health in the athlete population. Previous research has indicated that elite athletes may be under-reporting symptoms due to concerns of reported symptoms to have implications on their career (e.g., not making the team). It is also possible that gymnasts are more aligned with their body ideals and therefore report lower levels of eating disorder symptoms and mental health problems. This must be further addressed in future studies.

### Supplementary Information

Below is the link to the electronic supplementary material.

Supplementary Material 1.

Supplementary Material 2.

## Data Availability

Data for the current study can be reviewed upon request.
