# Supplementary material for: Assessment of eating disorder symptoms, compulsive exercise, body dissatisfaction and depression in Swedish national team gymnasts, with a one-year follow-up
Source: Eat Weight Disord. 2024 Jul 12;29(1):46. doi: 10.1007/s40519-024-01667-3 (PMC11245422; doi:10.1007/s40519-024-01667-3)
Supplement: Supplementary file 1 — Supplementary Material 1. [file 40519_2024_1667_MOESM1_ESM.docx]

**Supplement A**

Short description of the four disciplines in competitive gymnastics

**Men’s Artistic Gymnastics** (<https://www.gymnastics.sport/site/pages/disciplines/mag-presentation.php>):

“Gymnasts compete in six separate events: Floor Exercise, Pommel Horse, Still Rings, Vault, Parallel Bars and Horizontal Bar. Floor Exercise features tumbling and other acrobatic elements on a specially created Floor Exercise mat. Pommel Horse requires gymnasts to show circles, flairs and other swinging elements above the horse. The Still Rings is a test of endurance and strength, withe gymnasts supporting themselves and performing complex holds with their arms. Vault, one of two power events along with Floor Exercise, is the fastest event and features a gymnast propelling themselves over a vaulting table at full sprint. Parallel Bars and Horizontal Bar are both swinging events with frequent releases and a big dismount.

On all events, gymnasts are judged on difficulty of the exercise, execution, along with dynamics, including height and distance from apparatus, and must show strength, flexibility and balance”.

**Women’s Artistic Gymnastics** (https://www.gymnastics.sport/site/pages/disciplines/wag-presentation.php):

“Gymnasts perform on four apparatus - Vault, Uneven Bars, Balance Beam and Floor Exercise - with emphasis on agility, artistry, flexibility, power and style. Vault is the fastest event and features a gymnast propelling themselves over a vaulting table at full sprint, flipping or twisting back onto the mat. Uneven bars involves gymnasts performing swinging elements around two asymmetric bars, frequently releasing the bar and recatching it, while Balance Beam involves tumbling, acrobatics, choreography and dance elements on a narrow beam only 10 cm wide. Floor Exercises is the only event set to music and features tumbling, leaps, turns and choreography on a spring-loaded mat. On all events, gymnasts are judged on difficulty of the exercise, execution, along with dynamics, including height and distance from apparatus, and must show strength, flexibility, balance and rhythmic”.

**Rythmic Gymnastics** (https://www.gymnastics.sport/site/pages/disciplines/rg-presentation.php):

“Heavily influenced by ballet and modern dance, Rhythmic Gymnastics is the juncture of sport and art. Performing routines with music, either as individuals or in groups, rhythmic gymnasts execute maneuvers with hand-held apparatus: Hoop, Ball, Clubs, Ribbon and Rope.

Flexibility and musical interpretation are important elements in a Rhythmic exercise. However, it is the amount of risk a gymnast takes, often throwing the apparatus several meters into the air and losing sight of it while performing stunning leaps, turns or acrobatic maneuvers before regrasping it - that sets the routines apart”.

**Team Gym** (https://www.europeangymnastics.com/discipline/teamgym/intro):

“Team Gym is a team competition being performed in three disciplines: Floor, Tumble and Trampet. All three disciplines require effective teamwork and excellent technique with difficult skills in acrobatic and gymnastic elements. TeamGym competitions are divided into three sections: Women, Men and Mixed teams. A team may consist of 8 - 10 gymnasts, all of whom must compete on floor. On tumble and trampet, 6 gymnasts perform in each round.  In all disciplines, all the performing gymnasts are judged. A mixed team must consist of 50% male and 50% female gymnasts”.

**Trampoline** (https://www.gymnastics.sport/site/pages/disciplines/app-tra.php):

“Trampoline gymnasts compete in one of four categories: Individual Trampoline, Synchronised Trampoline, DoubleMini-trampoline and Tumbling. The best gymnasts often specialise in two of the four disciplines: Individual trampolinists often compete in Synchro as well, while some top Double Mini gymnasts take part in Tumbling at the World level. A trampoline is a device consisting of a bed, strong fabric stretched between a steel frame using many coiled steel springs. Gymnasts bounce on trampolines nearly 30 feet in the air performing 10 skills in sequence and without stops. The performance of the most complex movements, without deviations from the centre of the bed and at the highest point is the main objective of a trampoline routine”.
