# Supplementary material for: Assessment of eating disorder symptoms, compulsive exercise, body dissatisfaction and depression in Swedish national team gymnasts, with a one-year follow-up
Source: Eat Weight Disord. 2024 Jul 12;29(1):46. doi: 10.1007/s40519-024-01667-3 (PMC11245422; doi:10.1007/s40519-024-01667-3)
Supplement: Supplementary file 2 — Supplementary Material 2. [file 40519_2024_1667_MOESM2_ESM.docx]

**Supplement B**

The equation for the model where the independent variables were regressed simultaneously on body dissatisfaction, without controlling variables, is given by:

$${BD}_{i^{j}}=ß_{0}+ß_{1}*{DT}_{i^{j}}+ß_{2}*{EWC}_{i^{j}}+ß_{3}*{ARDB}_{i^{j}}+ß_{4}*{MADRS\text{-}S}_{i^{j}}+b_{i}+\varepsilon_{i^{j}}$$

Where:

- ${BD}_{i^{j}}$ is the Body Dissatisfaction score for the i-th subject at the j-th measurement.
- ${DT}_{i^{j}}$, ${EWC}_{i^{j}}$, ${ARDB}_{i^{j}},$ and ${MADRS\text{-}S}_{i^{j}}$ are the values for the independent variables Drive for Thinness, Exercise Weight Control, Avoidance and Rule Driven Behavior, and Montgomery-Åsberg Depression Rating Scale-Self resp. for the i-th subject at the j-th measurement.
- *ß_0_, ß_1_, ß_2_, ß_3_,* and *ß_4_* represents the coefficients for the fixed effects: intercept, Drive for Thinness, Exercise Weight Control, Avoidance and Rule Driven Behavior, and Montgomery-Åsberg Depression Rating Scale-Self.
- *b_i_* is the subject-specific intercept which accounts for the individual variability.
- $\varepsilon_{i^{j}}$ is the residual error term for the i-th subject at the j-th measurement.
- *i* ranges from 1 to 94, and *j* is 1 for baseline measurement and 2 for follow up measurement.

Results from the linear mixed models are presented as the ß-coefficient (95% CI). Analysis was carried out using R version 4.2.1 [34].
